# Analysis of homozygosity disequilibrium using whole-genome sequencing data

**DOI:** 10.1186/1753-6561-8-S1-S15

**Published:** 2014-06-17

**Authors:** Hsin-Chou Yang, Han-Wei Li

**Affiliations:** 1 Institute of Statistical Science, Academia Sinica, Nankang 115, Taipei, Taiwan

## Abstract

Homozygosity disequilibrium (HD), a nonrandom sizable run of homozygosity in the genome, may be related to the evolution of populations and may also confer susceptibility to disease. No studies have investigated HD using whole genome sequencing (WGS) analysis. In this study, we used an enhanced version of Loss-Of-Heterozygosity Analysis Suite (LOHAS) software to investigate HD through analysis of real and simulated WGS data sets provided by Genetic Analysis Workshop 18. Using a local polynomial model, we derived whole-genome profiles of homozygosity intensities for 959 individuals and characterized the patterns of HD. Generalized estimating equation analysis for 855 related samples was performed to examine the association between patterns of HD and 3 phenotypes of interest, namely diastolic blood pressure, systolic blood pressure, and hypertension status, with covariate adjustments for age and gender. We found that 4.48% of individuals in this study carried sizable runs of homozygosity (ROHs). Distributions of the length of ROHs were derived and revealed a familial aggregation of HD. Genome-wide homozygosity association analysis identified 5 and 3 ROHs associated with diastolic blood pressure and hypertension, respectively. These regions contain genes associated with calcium channels (*CACNA1S*), renin catalysis (*REN*), blood groups (*ABO*), apolipoprotein (*APOA5*), and cardiovascular diseases (*RASGRP1*). Simulation studies showed that our homozygosity association tests controlled type 1 error well and had a promising power. This study provides a useful analysis tool for studying HD and allows us to gain a deeper understanding of HD in the human genome.

## Background

Homozygosity disequilibrium (HD) describes a phenomenon in which a nonrandom pattern is observed for a sizable run of homozygosity (ROH) in the human genome, where ROH indicates a contiguous set of homozygous genotypes in an intact genomic region or allows to be interrupted by a small proportion of heterozygous genotypes arising from genotyping errors, missing genotypes, or mutations [1]. HD can result from autozygosity [2], natural selection [3], and chromosomal aberrations [4]. Previous studies suggested that HD may confer susceptibility to neurodevelopment-related disorders [5,6] and autoimmune diseases [1,7]. No studies have investigated HD with whole genome sequencing (WGS) analysis. This study analyzed a real human WGS data set and simulated data sets provided by Genetic Analysis Workshop 18 (GAW18) with four major aims. The first aim was to develop statistical methods and analysis tools to examine HD in WGS data. The second aim was to characterize patterns of HD in the human genome. The third aim was to identify regions of HD associated with diastolic blood pressure (DBP), systolic blood pressure (SBP), and hypertension status. The final aim of this study was to evaluate the performance of the proposed genome-wide homozygosity association analysis approach on the simulated data set. This study constitutes a useful resource for examining HD and provides insight into the potential roles of HD in population genetics and medical genetics.

## Methods

### Materials

GAW18 provided a combined imputation data set derived from deep sequencing data for the whole genomes of 464 individuals and genome-wide association genotype data for 495 individuals. All 959 individuals were from 20 large independent pedigrees enrolled in the T2D-GENES (Type 2 Diabetes Genetic Exploration by Next-generation sequencing in Ethnic Samples) Project 2. Full information about blood pressures (DBP and SBP) and covariates (age and gender) was also available for 855 of these individuals. In this study, an individual was considered hypertensive if he or she had ever taken antihypertensive medication or his or her DBP was greater than 90 mm Hg or SBP was greater than 140 mm Hg at the most recent examination. The genome of each individual was sequenced by Complete Genomics with an average depth of coverage of 60x. Multiple quality control procedures were carried out to filter out single-nucleotide variants (SNVs) with poor performance in allele balance, strand bias, fraction of bases with low quality, and Mendelian errors by GAW18. The WGS data set of 464 individuals contained 24 million of SNVs that passed quality filters, and more than 51% of them were rare variants (RVs). The combined data set of 959 individuals contained 8,348,663 single-nucleotide polymorphisms (SNPs) and 5,573,886 RVs for odd-numbered autosomes. In addition, GAW18 provided 200 simulation data sets of quantitative trait Q1. Q1 was generated from a normal distribution and was independent of genetic variants in this study.

### Statistical methods

For this study, we developed an enhanced version of the Loss-Of-Heterozygosity Analysis Suite (LOHAS) software [8]. LOHAS was originally developed for detecting loss of heterozygosity in cancer research and identifying long contiguous stretches of homozygosity in population genetics studies using SNP genotype data. LOHAS provides a two-step procedure for homozygosity association studies. First, LOHAS constructs sliding windows on a chromosome using a nearest neighbor method and estimates homozygosity intensity in each window for each individual using a local polynomial model. In the model, the homozygote-heterozygote status was regressed by the physical positions of SNVs to calculate the homozygosity intensity. Homozygosity intensities are values between 0 and 1. Second, LOHAS performs a linear-rank association test to identify runs of homozygosity (ROHs) with differential homozygosity intensities between the study groups.

We extended LOHAS to handle large numbers of SNPs and RVs in the WGS data by introducing the following adapted coding system of homozygote-heterozygote status. Major and minor alleles at a SNV were denoted *A *and *a*, respectively. SNVs were classified as SNPs if their minor allele frequency (MAF) was higher than 0.05 or as RVs if lower than 0.05, where MAF was calculated across the whole population. For the SNP analysis, the coding system was the same as that used in the original LOHAS software. Homozygotes (*aa *and *AA*) were coded as 1 and heterozygotes (*Aa*) as 0. For the RV analysis, rare homozygotes (*aa*) and heterozygotes (*Aa*) were coded as 1 and 0, respectively. Because common homozygotes (*AA*) of RVs are less informative and therefore could dilute the assessment of other informative homozygotes when defining ROH, they were analyzed separately. Homozygosity intensity of a window anchored at a common homozygote of a RV was estimated by inputting the physical position of the anchor into the fitted local polynomial model, which was built based on genotypes except for common homozygotes of RVs. We also expanded LOHAS software by adding model-based methods for studying a dichotomous or quantitative trait with or without an adjustment for covariates. A new analysis module of generalized estimating equation (GEE) was included for analysis of related individuals, and a new analysis module of linear regression model was included for analysis of unrelated individuals.

To estimate whole-genome homozygosity intensities of all 959 individuals, a window size, 5% of SNVs on a chromosome, was considered. Each window contained 11,968 to 65,465 SNVs. The GEE was used to analyze 855 related samples with WGS data, blood pressures, hypertension status, and covariates. Homozygosity intensities were modeled as a continuous response to correlate with DBP values, SBP values, and hypertension status, with concomitant adjustment for age and gender.

We also evaluated type 1 error and test power of the homozygosity association tests using simulation data. Type 1 error was analyzed by examining the association between homozygosity intensities and Q1 (with covariate adjustments for age and gender) using the GEE analysis. We calculated the proportion of times that the null hypothesis was rejected over 200 simulations at each window and then calculated the average type 1 error over all windows. Test power was analyzed by choosing 3 regions on chromosome 21 containing 200 SNVs for each with varying proportions of RVs. The 3 regions contained 51.5%, 66.5%, and 80.0% RVs representing the 10th, 50th, and 90th percentiles of proportions of RVs on chromosome 21, respectively. The 3 regions were designated as Q1-associated ROHs by a logistic regression model as follows:

$$\text{Logit}\left(p\right)=b_{0}+b_{1}\cdot \text{ Q}1,$$

where *p *is the probability that all genotypes in the region were replaced by homozygotes and *b*_0 _and *b*_1 _are the regression coefficients. The test power was calculated as the proportion of times that the null hypothesis was rejected over 200 simulations at a Q1-associated ROH.

## Results

**Figure 1:**
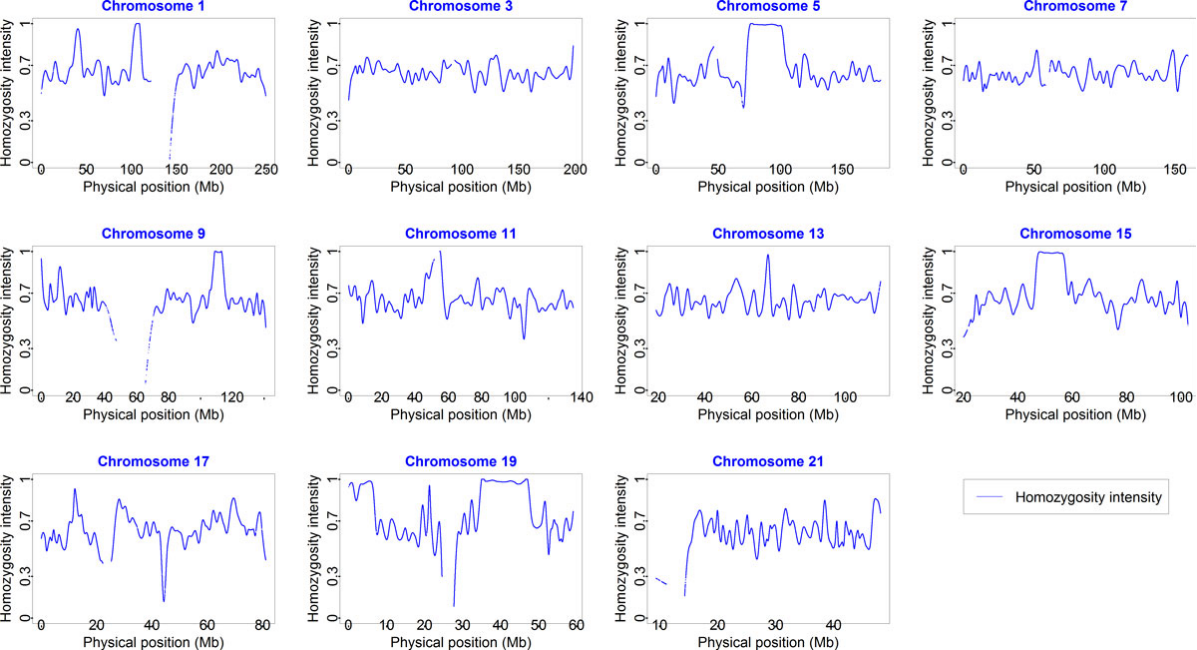
**Whole-genome profiling of homozygosity intensity on odd-numbered chromosomes for subject T2DG0300128 in pedigree 3**. We used a window size of 5% of single-nucleotide variants (SNVs) on each chromosome. The homozygosity intensity curve of a male individual is shown. Each vertical axis represents homozygosity intensity from 0 to 1, and each horizontal axis represents the physical positions (Mb) of anchor SNVs of the runs of homozygosity.

Our analyses were performed without knowledge of the underlying simulation model. Whole-genome profiles of homozygosity intensities for 959 individuals were derived from the GAW18 data set. Approximately 4.48% of individuals (43/959) carried sizable ROHs with homozygosity intensities greater than 0.9 and run lengths greater than 5 Mb. The minimum, 25th, 50th, and 75th percentiles and maximum of ROH lengths were 5.07, 5.66, 7.40, 13.61, and 44.60 Mb, respectively. The minimum, 25th, 50th, and 75th percentiles and maximum of total lengths of ROHs carried by an individual were 5.07, 5.64, 7.54, 14.81, and 105.85 Mb, respectively. Figure 1 shows an example of a male subject (T2DG0300128) who carries multiple ROHs on chromosomes 1, 5, 9, 11, 13, 15, and 19. We also observed a familial aggregation of ROHs. For example, four individuals (T2DG2701096, T2DG2701097, T2DG2701098, and T2DG2701099) in pedigree 27 carried the identical ROHs on chromosomes 13 and 21.

**Figure 2:**
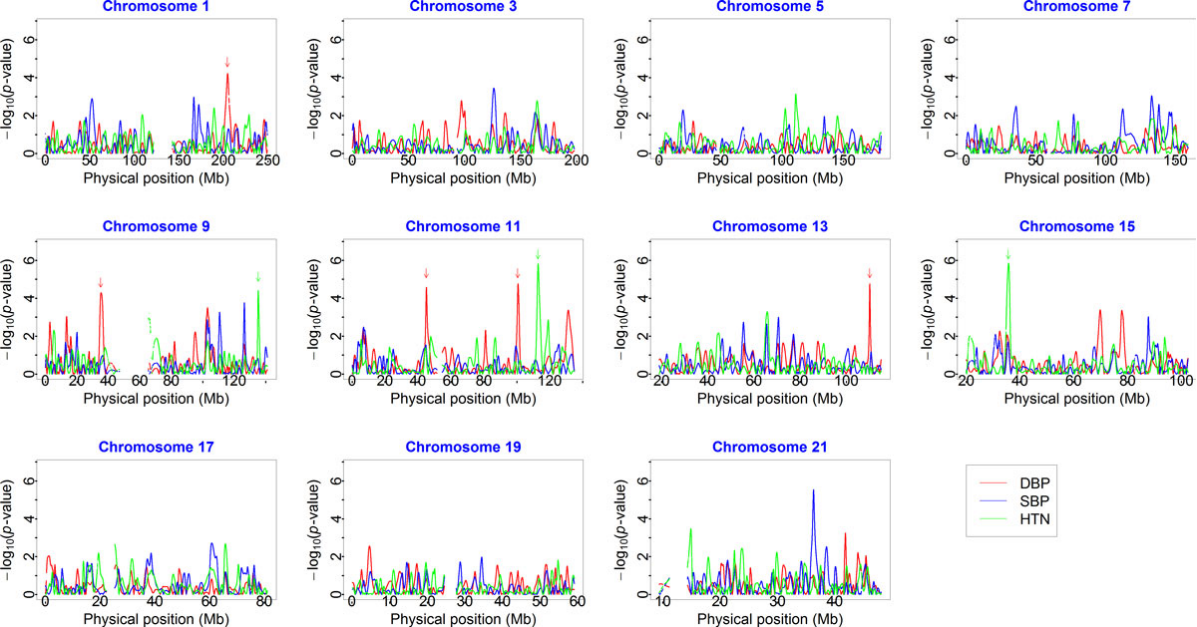
**Examination of the association between homozygosity intensities on odd-numbered chromosomes and 3 phenotypes of interest**. We used a window size of 5% of single-nucleotide variants (SNVs) on each chromosome. Homozygosity association tests for diastolic blood pressure (DBP) (red line), systolic blood pressure (SBP) (blue line), and hypertension (HTN) status (green line) are shown. Each vertical axis represents the raw *p*-values (-log_10 _scale) of the association tests (generalized estimating equation model), and each horizontal axis represents the physical positions (Mb) of anchor SNVs of runs of homozygosity. The arrows point to *p*-values that were significant after controlling for false discovery rate.

We carried out whole-genome homozygosity tests to identify DBP-, SBP-, or hypertension-associated ROHs. GEE analysis of 855 related individuals identified several DBP- or hypertension-associated ROHs after controlling for false discovery rate (Figure 2). DBP-associated ROHs were located in the regions ranged from 198.4 to 210.0 Mb on chromosome 1 (adjusted *p*-value = 0.0396), from 31.9 to 38.1 Mb on chromosome 9 (adjusted *p*-value = 0.0396), from 41.4 to 48.5 Mb (adjusted *p*-value = 0.0396) and from 97.7 to 103.3 Mb (adjusted *p*-value = 0.0396) on chromosome 11, and from 108.0 to 112.1 Mb on chromosome 13 (adjusted *p*-value = 0.0396). Hypertension-associated ROHs were located in the regions ranged from 132.7 to 137.9 Mb on chromosome 9 (adjusted *p*-value = 0.0264), from 109.5 to 116.1 Mb on chromosome 11 (adjusted *p*-value = 0.0050), and from 33.7 to 37.5 Mb on chromosome 15 (adjusted *p*-value = 0.0050). No SBP-associated ROHs were found.

We evaluated type 1 error of the homozygosity association tests by analyzing 200 simulation data sets of quantitative trait Q1. For a GEE association analysis of the 855 related individuals, the mean of type 1 errors was 0.03. We also evaluated the test power of our homozygosity association tests by analyzing the 200 simulation data sets with 3 Q1-associated ROHs created under the parameters that *b*_0 _= −20 and *b*_1 _= 0.5 in our simulation model. For the GEE analysis of 855 related individuals, the power for the 3 Q1-associated ROHs containing 51.5%, 66.5%, and 80.0% RVs was 1.00, 1.00, and 1.00, respectively. The power was reduced to 0.910, 0.975, and 0.945 when *b*_0 _= −20 was changed to *b*_0 _= −10.

## Discussion and conclusions

This study has made several contributions to WGS data analysis of HD in the human genome. First, we adapted LOHAS software to enable more complete analysis of WGS data for HD. Two new homozygosity association analysis modules, a regression-based analysis for unrelated individuals and a GEE-based analysis for related individuals, have been incorporated into LOHAS. Software, examples, and user guide are now available free of charge at http://www.stat.sinica.edu.tw/hsinchou/genetics/loh/LOHAS.htm.

Second, we characterized the patterns of HD by analyzing a large WGS data set of 959 individuals from 20 pedigrees. The distribution of lengths for sizable ROHs and a distribution of total run length for one individual were obtained. The larger individual total run lengths of an individual may suggest autozygosity owing to inbreeding, consanguineous marriage, or a recent common ancestor. This approach may also be useful in identifying specific types of chromosomal aberrations such as uniparental disomy, hemizygous deletion, and loss of heterozygosity in cancer studies. In addition, we found that patterns of HD differ among individuals. We also observed familial aggregation, suggesting a genetic component of HD. A sensitivity analysis, which considered 3 thresholds of MAF for defining RVs (MAF = 0.04, 0.05, and 0.06), showed that the results were robust to a small change in the threshold of MAF for defining RVs. These findings suggest potential applications of these methods in genetic studies.

Third, our GEE-based homozygosity association tests identified 5 DBP-associated ROHs and 3 hypertension-associated ROHs. Genes in these ROHs include some that are associated with calcium channels, renin catalysis, insulin-like growth factor (IGF), blood groups, apolipoprotein, cardiovascular diseases, and rat sarcoma. Specifically, within the DBP-associated ROHs, two genes on chromosome 1q32 encode proteins that inactivate calcium channels in skeletal muscle cells (*CACNA1S*) and play a role in renin catalysis (*REN*), both of which are important for the regulation of blood pressure. *IGFBPL1 *on 9p13.1 encodes IGF binding protein. Within the hypertension-associated ROHs, *ABO *on 9q34.2 is associated with the ABO blood group system. *APOA1, APOA4, APOA5*, and *APOC3 *on 11q23-q24 encode apolipoproteins and have been associated with cardiovascular diseases. *RASGRP1 *on 15q14 encodes a protein that functions as a Ras activator as well as a switch that is regulated by calcium and diacylglycerol, and guanosine triphosphate-guanosine diphosphate exchange. However, these results are still preliminary, and the findings should be further investigated in future studies.

We also examined homozygosity associations of 142 samples from unrelated individuals (85 hypertensive cases and 57 normotensive control participants) using a linear regression analysis. However, probably owing to the relatively small number of samples, none of the ROHs in this analysis was found to be associated with DBP, SBP, or hypertension after controlling for false discovery rate.

In summary, the enhanced LOHAS developed in this study represents a useful tool for studying HD under different ascertainment schemes (unrelated and related individuals), phenotypes of interest (dichotomous disease status and quantitative traits), and experimental platforms (SNP microarrays and next-generation sequencing experiments). Performance of the improved LOHAS homozygosity association tests was evaluated by simulation studies, and the results suggest that the methods are reliable with a high test power and well-controlled type 1 error. Additional simulation studies and real data analyses will further elucidate the limitations of our methods and helps us further understand HD in the human genome.

## Competing interests

The authors declare that they have no competing interests.

## Authors' contributions

HCY conceived of the study, developed statistical methods, and prepared the manuscript. HWL developed analysis software and analyzed the data with HCY. Both authors read and approved the final manuscript.
